# Correlation of malocclusion and mouth breathing rates from a novel monitor

**DOI:** 10.1007/s00784-025-06709-3

**Published:** 2026-01-07

**Authors:** D. N. Li, Y. Z. Tu, Z. Lu, L. Mei, F. Hua, M. H. Li, R. B. Zhu, H. He, Y. Luo, K. Qi

**Affiliations:** 1 Key Laboratory of Shaanxi Province for Craniofacial Precision Medicine Research, College of Stomatology, Xi’an Jiaotong University, 98 XiWu Road, Xi’an, 710004 China; 2 Mirco and Nano-Technology Research Center, State Key Laboratory for Manufacturing Systems Engineering (Xi’an Jiaotong University), Xi’an, 710049 China; 3 Discipline of Orthodontics, Department of Oral Sciences, Faculty of Dentistry, University of Otago, 310 Great King Street, Dunedin, 9016 New Zealand; 4 Center for Orthodontics and Pediatric Dentistry at Optics Valley Branch, School & Hospital of Stomatology, Wuhan University, Wuhan, China; 5 Department of Orthodontics, School & Hospital of Stomatology, Wuhan University, Wuhan, China

**Keywords:** Humidity, Capacitance, Reliability, Three-dimensional imaging, Cone-beam computed tomography

## Abstract

**Objective:**

This study aimed to develop, validate, and implement a novel wearable monitor to detect mouth breathing rates and analyze their correlation with malocclusion severity using craniofacial measurements obtained from CBCT and 3D photographs.

**Materials and methods:**

The monitor was equipped with two sensors mounted in a 3D-printed holder for oral and nasal breathing detection respectively, which senses humidity changes by silk fibroin and interprets into capacitive values. After qualitatively and quantitatively validated by polysomnography and the infrared thermography, the monitor was employed to calculate mouth breathing rates. Malocclusion parameters were derived from CBCT and 3D photographs, and correlated to mouth breathing rates.

**Results:**

The accuracy, precision and recall of this novel monitor were 101.07 ± 5.21%, 98.80 ± 3.70% and 99.85 ± 2.08% respectively. The temperature changes and normalised capacitance values showed strong negative correlation during both inhalation and exhalation, with the mean correlation coefficients K at -0.8985 and -0.9332 respectively in the same breathing process. Strong correlations were identified between mouth breathing rates and maxillary canine width, palatal operculum height, left and right mandibular angle, lower face height, lower lip protrusion, nasolabial angle, chin-lip angle and the volumes of adenoid, nose, total airway and nasopharynx airway.

**Conclusion:**

This novel wearable monitor can qualitatively and quantitatively monitor mouth and nasal breathing accurately and precisely, potentially establishing itself as gold standard for mouth breathing diagnose and severity assessment. The strong correlations observed between mouth breath rates and malocclusion emphasize the importance of early orthodontic intervention in addressing mouth breathing habits.

**Supplementary Information:**

The online version contains supplementary material available at 10.1007/s00784-025-06709-3.

## Introduction

Mouth breathing is defined as over 25% − 30% of the air passing through the mouth instead of the nose [1]. It is usually caused by upper airway obstruction, such as adenoid hypertrophy, allergic rhinitis, enlarged tonsils, and deviated nasal septum [2–4]. Mouth breathing leads to "adenoid facies" characterized by underdevelopment of the upper lip, posterior displacement of the hyoid bone and a series of malocclusions, including a narrow or "V" shaped upper dental arch, increased height of the anterior portion, increased angle of the mandibular plane, and posterior rotation of the mandible [5–9]. These have been validated by both two-dimensional (2D) and three-dimensional (3D) [10, 11] analyses, however, quantitative evaluation on the correlation between mouth breathing rates and malocclusion severity, especially 3D quantification, has not been investigated so far.

However, there is a lack of effective devices for mouth breathing diagnosis. The most prevalent clinical method to identify mouth breathing is the combination of identifying “adenoid facies” and using questionnaires, along with parental reports of nocturnal mouth breathing [12], which is prone to recall bias. The fog mirror test could also be used [13], but it is subject to interference from nasal breathing, and prolonged monitoring is impractical[14]. Moreover, polysomnography (PSG) can assess oro-nasal breathing simultaneously [15], but it requires high costs and healthcare professionals to apply properly [16, 17]. PSG requires wearing it overnight in a ward. The unfamiliar clinical environment may lead to reduced sleep quality in children, and it is difficult to ensure their cooperation throughout the recording process. These factors may result in suboptimal or non-representative sleep data[18]. Overall, current diagnostic methods are often indirect, subjective, or impractical, and there is a need for an accurate and wearable monitor for clinical quantitative assessment of mouth breathing.

Oral and nasal breathing can induce local humidity changes around the mouth and nose. Thus, theoretically, measuring oral and nasal respiration through the analysis of resistive or capacitive parameters of humidity-sensitive materials is a viable approach. In our previous research, we successfully developed an innovative capacitive humidity sensor utilizing silk fibroin (SF), which exhibits rapid response and high biocompatibility [19]. The dielectric constant of modified SF films responds effectively to vapor penetration during breathing, enabling the sensor to differentiate between various intensities and rates of respiration at an ultra-fast response rate of 4 Hz, marking it as one of the most sensitive options for breathing detection.

**Fig. 1:**
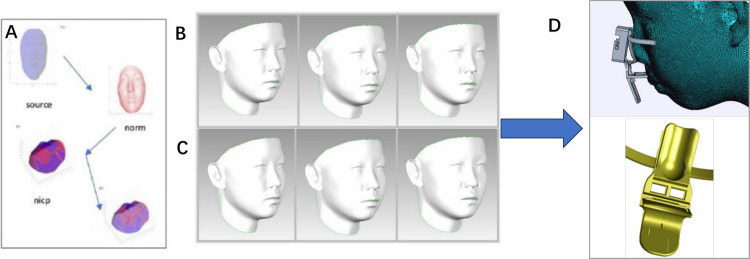
Construction of the “average face model” and design of the monitor holder: **a**) LSFM model construction process. **b**) Average face model with different sagittal face types for males (skeletal class-I, II and III from left to right). **c**) Average face model with different sagittal face types for females (skeletal class-I, II and III from left to right). **d**) Design of the monitor based on the average face model

**Fig. 2:**
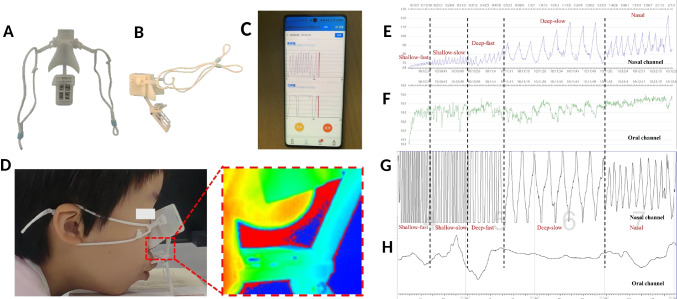
Qualitatively and quantitatively experimental validation of the mouth breathing monitor. **a**) Front view of the mouth breathing monitor. **b**) Side view of the mouth breathing monitor. **c**) The application on the smartphone records the interface of breathing. **d**) Volunteers wearing mouth breathing monitor under infrared thermography

**Fig. 3:**
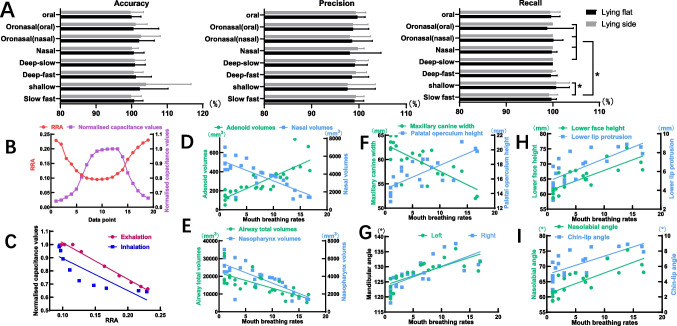
Qualitative and quantitative results of the mouth breathing monitor; Pearson ‘s correlation coefficients between the 3D photographs measurements, CBCT measurements and mouth breathing rates. **a**) Histograms of accuracy, precision and recall of the number of breathing measured by mouth breathing compared to PSG(*, p<0.05) ; **b**) Breathing curves plotted by infrared thermography and mouth breathing monitor during the same nasal breathing process; **c**) Linear equations of one volunteer between RRA and normalised capacitance values during exhalation and inhalation; **d**) Pearson‘s correlation coefficients between mouth breathing rates and adenoid volumes and nasal volumes (K= 0.838/-0.609); **e**) Pearson‘s correlation coefficients between mouth breathing rates and airway total volumes and nasopharynx volumes (K= -0.705/-0.784); **f**) Pearson‘s correlation coefficients between mouth breathing rates and maxillary width and palatal operculum height (K= -0.762/0.748); **g**) Pearson‘s correlation coefficients between mouth breathing rates and left and right mandibular angle (K= 0.742/0.741); **h**) Pearson‘s correlation coefficients between mouth breathing rates and lower face height and lower lip protrusion (K= 0.786/0.734); **i**) Pearson‘s correlation coefficients between mouth breathing rates and nasolabial angle and chin-lip angle (K= -0.795/0.773)

The objective of this study is to qualitatively evaluate mouth breathing, and its correlation with malocclusion. A novel monitor based on humidity sensitivity was developed and validated. Subsequently, we measured the mouth breathing rates and analyzed their correlation with three-dimensional (3D) malocclusion parameters obtained from cone-beam computed tomography (CBCT) and 3D photographs.

## Materials and methods

### Study design

Ethics of the study was approved by the Institutional Review Board, Xi’an Jiaotong University (2023-XJKQIEC-QT-0019–002). The study follows the STROBE Guidelines for observational human research. Written informed consent was obtained from each participant prior to the commencement of the study.

A personalized mouth breathing monitor was developed and qualitatively compared to standard polysomnography (PSG). Diagnostic accuracy tests were performed. For quantitative validation, infrared thermography was employed. Furthermore, the correlation coefficient between mouth breathing rates and three-dimensional indicators of malocclusion obtained by CBCT and 3D photographs were analyzed. This study was conducted in the Hospital of Stomatology, Xi’an Jiaotong University.

### Equipment for monitor validation

In order to construct a customized sensor holder, a total of 677 participants (400 girls, mean age 12.41 ± 1.52 years; 277 boys, mean age 12.61 ± 1.40 years) were recruited to develop an "average face model". This model was generated using the Large Scale Facial Models (LSFM) algorithm. The participants were classified by both skeletal classification and sex, resulting in the creation of six distinct average face models using the LSFM approach.

The mouth-breathing monitor was equipped with two capacitive humidity sensors positioned to detect humidity changes near the nose and mouth respectively at a frequency of 10 Hz. These sensors were integrated into a custom-made capacitance measurement module and powered by a miniature lithium battery, ensuring its suitability for real-time and wireless monitoring (Fig. 2A-B) [20]. Breathing data were transmitted via Bluetooth to a smartphone application and uploaded to a website for further analysis (Fig. 2C).

To evaluate the potential effect of the distance between the monitor and nostrils on capacitance values during breathing, a preliminary study was conducted. The results showed no statistically significant difference between distances of 10 mm, 15 mm, and 20 mm, allowing for the holder design to remain within this range (Supplementary Fig. 1).

For qualitative comparison with polysomnography (PSG), volunteers wore both devices simultaneously. Nasal and oral breathing were recorded using PSG through a nasal pressure transducer and an oral thermal airflow sensor (thermistor) respectively. The PSG data were processed and displayed using Sleepware G3 (version 3.9.6.0) on a computer.

For quantitative validation, volunteers wore the monitor in front of an infrared thermography camera, which recorded temperature changes caused by breathing [21, 22] (Fig. 2D). The videos were synchronized with the breathing data captured by the monitor and compared for evaluation.

### Participants for monitor validation

A sample size of 26 participants (14 girls and 12 boys, mean age 9.89 ± 1.61 years) was calculated to achieve an accuracy of 100 ± 10%, using α = 0.05, power (P) = 0.8 and determined by G*Power (version 3.1). To conduct the quantitative experiment with polysomnography (PSG), 28 volunteers were recruited from the Stomatology Hospital of Xi'an Jiaotong University. Additionally, 10 volunteers participated in the qualitative analysis, to test 10 produced monitors using infrared thermography.

Volunteers first confirmed that they experienced no discomfort while using both the PSG and the monitor, followed by a brief pre-test lasting 30 s to ensure proper preparation. Each volunteer was asked to breathe in two positions: supine (lying flat) and lateral (lying on the side). For each position, seven distinct breathing patterns were tested: shallow-fast breathing, shallow-slow breathing, deep-fast breathing, deep-slow breathing, nasal breathing, oronasal breathing, and oral breathing in a calm state [23]. For each breathing pattern in each posture, at least five complete respiratory waveforms were recorded. Due to the limitations of infrared thermography, quantitative analysis was performed while the volunteers were seated.

### Qualitative and quantitative validation of the monitor

Qualitative analysis was performed by comparing the breathing recorded by the monitor with that of PSG (Fig. 2E-H). The monitor's algorithm detected respiration when the change of capacitance value exceeded a fixed threshold of 3. The following three parameters were used. Accuracy = (TP + FP)/(TP + FN) (%); Precision = TP/(TP + FP) (%); Recall = TP/(TP + FN) (%) [24]. True Positive (TP) indicates instances where both the mouth-breathing monitor and PSG detected respiration. False Positive (FP) indicates instances where the mouth-breathing monitor detected respiration but PSG did not. False Negative (FN) indicates instances where PSG detected respiration but the mouth-breathing monitor did not.

The accuracy, precision, and recall of the monitor were evaluated respectively across different breathing patterns during both flat-lying and side-lying positions.

Infrared thermography was used to quantitatively validate the monitor. A relatively enclosed space was created between the nose and the monitor, allowing infrared thermography to detect temperature changes within this space. These changes were analyzed by calculating the ratio of the red area (RRA), representing heat, to the total area using Image-Pro Plus 6.0. The RRA curve, generated using GraphPad Prism 10, depicted real-time temperature variations caused by respiration. Simultaneously, the monitor detected breathing via capacitance value changes, which were normalized and analyzed via a web interface. The linear correlation between RRA and normalized capacitance values for the same breathing process was analyzed using MATLAB (version 23.2.0).

### Participants for craniofacial measurements

Inclusion criteria for those who underwent CBCT and 3D photographs were as follows: 1) parents reporting children with mouth breathing for more than 2 years; 2) aged 6–12 years; 3) BMI in the normal range (18.5–23.9); 4) presence of adenoidal hypertrophy; 5) presence of narrow dental arches or adenoidal facies. The following four categories were excluded from the study based on the literature [25]: 1) long-term use of topical or systemic corticosteroids, nasal vasoconstrictors, muscle relaxants, and/or barbiturates within the past 3 months; 2) experienced a respiratory tract infection within the past 3 weeks or those at risk of allergies; 3) history of chronic obstructive pulmonary disease, cardiovascular disease, craniofacial anomalies, or neurological disorders that could impede cooperation with the examination; 4) history of chest, abdominal, and/or nasal surgery.

### Acquisition of craniofacial data

All CBCT scans were performed using the same procedure with the same equipment (i-CAT, Imaging Sciences International, Hatfield, PA, USA) (120 kV,5 mA, 14 *17 cm FOV, 0.4 mm voxel, and scan time of 8.9 s). Patients were asked to sit erectly and hold their breath at the end of expiration, with their head in a natural posture, their jaw in maximal intercuspation, and not to swallow. All digital image data were recorded in Digital Imaging and Communications in Medicine (DICOM) format. 3D photographs were captured via the 3dMD face system (3dMD, Atlanta, GA, USA) in an ordinary indoor environment under incandescent lighting. The area from subjects' hairline to thyroid cartilage level was unobstructed, with the forehead and both ears fully exposed. Subjects sat upright with straight upper bodies, maintaining a natural head position, relaxed facial muscles, naturally closed lips, light occlusal contact of bilateral posterior teeth, no expressions, and forward gaze. They were positioned ~ 95 cm from the 3dMD binocular components, with chair height adjusted to align the head with the components. Shooting time was ~ 0.5 ms, and images were exported and saved in obj format. One 3D photograph was taken for each patient. The 3D airway parameters were measured by Dolphin Imaging software version 11.7. (Dolphin Imaging & Management Solutions, Chatsworth, CA, USA).

### Measurements of craniofacial data

A total of 36 CBCT measurements of 28 patients (16 girls and 12 boys, mean age 9.69 ± 1.50 years) were measured by a calibrated investigator with Dolphin 11.7 (Dolphin Imaging & Management Solutions, Chatsworth, CA, USA) [26–30]. In airway measurements, the patient's head was positioned with the palatal plane parallel to the horizontal plane in the sagittal view and centered along the coronal and axial axes, establishing a reference plane for standardizing all scans before measurement. Subsequently, the optimal parasagittal view of the airway, which clearly shows the posterior nasal spine and the second cervical vertebra, was identified. The airway was localized by examining consecutive volume slices and placing seed points in the corresponding image voids; the software then automatically selected adjacent empty regions to recognize the airway. The lower boundary of the oropharynx is a plane parallel to the Frankfort horizontal (FH) plane, located at the anterior inferior edge of the third cervical vertebra, while that of the laryngopharynx, also parallel to the FH plane, is at the anterior inferior edge of the fourth cervical vertebra (Supplementary Figs. 2–5). The Geomagic Design X was used to analyze a total of 52 measurements, including 44 linear and 8 angular measurements obtained from 3D photographs of 22 patients (16 girls and 6 boys, mean age 9.54 ± 1.55 years) [31, 32] (Supplementary Fig. 6). Patient information was blinded to the investigator.

### Statistical analysis

Statistical analyses were performed using IBM SPSS Statistics (version 22.0, IBM Corp, Armonk, New York, USA), with significance set at p < 0.05. The Kolmogorov–Smirnov test was used to assess the normality of the data distribution. If the data were not normally distributed, the Kruskal–Wallis test was applied to determine statistical differences among independent groups, with post-hoc analysis conducted using the Bonferroni correction method to compare differences between groups. Pearson correlation analysis with corresponding 95% confidence intervals (CIs) were used to examine the correlation between the above-mentioned outcome measurements and mouth breathing rates (*p* < 0.05). The process of image acquisition and analysis was carried out by the same examiner who was blinded to the group and time points of each image. An initial method error assessment was conducted to eliminate intra-operator variations. In this regard, eight randomly chosen CBCT and 3D photographs were done again after a two-week interval by the same operator and an additional operator, with the inter-and intraclass coefficients employed to determine the reproducibility of the procedure.

## Results

### Validation of the monitor

The average face model was generated and illustrated in Fig. 1. Sensor holders were 3D printed to match this model. The overall performance of the mouth-breathing monitor demonstrated high reliability, with accuracy, precision, and recall of 101.07 ± 5.21%, 98.80 ± 3.70%, and 99.85 ± 2.08%, respectively. Statistical analysis revealed no significant differences in the accuracy or precision across different breathing patterns when comparing the supine and lateral positions. Similarly, recall did not vary significantly between breathing patterns in the supine position, but varied in the lateral position. The recall for deep-fast breathing (99.17 ± 1.91%) was significantly lower than that for shallow-slow (100.71 ± 2.86%), deep-slow (100.00 ± 0.00%), nasal (100.00 ± 0.00%), oronasal (nasal, 100.00 ± 0.00%), and oronasal (oral, 100.00 ± 0.00%) breathing (p < 0.05) (Fig. 3A). No significant differences were observed between other breathing patterns in the lateral position. Additionally, posture did not significantly affect accuracy, precision, or recall for the same breathing pattern (Supplementary Table 1).

The mean correlation coefficient (K) across the ten volunteers was −0.8639. During inhalation and exhalation, the correlation coefficients were −0.8985 and −0.9332, respectively. The mean linear equations describing the relationship between the temperature changes (represented by the red area ratios from the infrared thermography) and the capacitance values from the mouth-breathing monitor were expressed by the following linear equations: for inhalation, Y = −2.6947X + 1.2196 (R^2^ = 0.8073), and for exhalation, Y = −2.232X + 1.1147 (R^2^ = 0.8710) (Fig. 3B-C).

After validating that the monitor can accurately detect mouth breathing, a study on the correlation between mouth breathing rates and malocclusion was conducted.

### Correlation of malocclusion and mouth breathing rates

The inter-and intraclass coefficients for the CBCT measurements ranged from 0.856 to 0.968 (P = 0.000.*) and 0.849 to 0.974 (P = 0.000.*). For the CBCT measurements, the volumes of adenoid had a strong positive correlation with mouth breathing rates (Pearson's r = 0.838) (Table 1, Fig. 3D). Conversely, the volumes of nose, total airway and nasopharynx airway were strongly negatively correlated with mouth breathing rates (Pearson's r = −0.609/−0.705/−0.784) (Table 1, Fig. 3D-E). Moreover, maxillary canine width had a strong negative correlation with mouth breathing rates (Pearson's r = −0.762) (Table 1, Fig. 3F). Additionally, palatal operculum height, left and right mandibular angle were strongly positively correlated with mouth breathing rates (Pearson's r = 0.748/0.742/0.741) (Table 1, Fig. 3F-G). The divergence of the mandible displayed a moderate negative correlation with mouth breathing rates (Pearson's r = −0.442) (Table 1).

**Table 1:** Pearson’s correlation coefficients and 95% CIs between the CBCT measurements and mouth breathing rates

	The CBCT measurements	Pearson's correlation coefficients	95% CIs
Line	Nasopharyngeal heights	0.178	(−0.279, 0.492)
	Oropharyngeal heights	0.089	(−0.386, 0.246)
	Laryngeal heights	−0.223	(−0.582, 0.105)
	Toltal heights	−0.033	(−0.594, 0.492)
	the length of the mid-palatal suture	−0.078	(−0.346, 0.428)
	Palatal length (PL)	0.059	(−0.337, 0.420)
	the thickness of the left alveolar crest	0.018	(−0.224, 0.489)
	the thickness of the right alveolar crest	0.249	(0.109, 0.823)
	maxillary canines width^a^	−0.762	(−0.894, −0.510)
	palatal operculum height^a^	0.748	(0.486, 0.887)
	Mandibular width	0.024	(−0.376, 0.727)
	CL.GoL.Me	−0.293	(−0.476, 0.726)
	CR.GoR.Me	−0.287	(−0.507, 0.726)
	CL-CR	−0.235	(−0.496, 0.379)
	GoL-GoR	0.198	(0.100, 0.649)
	ZL-ZR	0.223	(0.103, 0.721)
	CL-GoL	0.046	(−0.346, 0.497)
	CR-GoR	−0.123	(−0.436, 0.467)
	GoL-Me	−0.093	(−0.156, 0.449)
	GoR-Me	0.213	(0.160, 0.727)
	CL-A	0.198	(0.019, 0.492)
	CR-A	0.173	(−0.187, 0.498)
	CL-Gn	0.162	(−0.249, 0.725)
	CR-Gn	−0.143	(−0.426, 0.492)
Angle	Left mandibular angle^a^	0.742	(0.476, 0.884)
	Right mandibular angle^a^	0.741	(0.474, 0.883)
	Divergence of the mandible^a^	−0.442	(−0.728, −0.025)
Area	Minimum areas of nasopharynx	−0.098	(−0.349, 0.279)
	Minimum oropharyngeal areas	0.031	(−0.149, 0.379)
	Minimum laryngeal areas	−0.097	(−0.414, 0.249)
Volume	Nasal volumes^a^	−0.609	(−0.801, −0.306)
	Nasopharyngeal volumes^a^	−0.784	(−0.895, −0.580)
	Oropharyngeal volumes	0.090	(−0.423, 0.513)
	Laryngeal volumes	0.194	(−0.167, 0.480)
	Adenoid voulmes^a^	0.838	(0.676, 0.923)
	Total airway volume^a^	−0.705	(−0.853, −0.450)

^a^The correlation is significant when the confidence (2-tailed) is 0.01

The inter-and intraclass coefficients for the 3D photographs ranged from 0.851 to 0.962 (P = 0.000.*) and 0.868 to 0.968 (P = 0.000.*). For the 3D photographs measurements, there is a moderate positive correlation between the inferior facial height, lower lip protrusion and chin-lip angle and mouth breathing rates, with Pearson's correlation coefficients of r = 0.786, r = 0.734 and r = 0.773, respectively (Table 2, Fig. 3H-I). Conversely, the nasolabial angle was strongly negatively correlated with mouth breathing rates (Pearson's r = −0.795) (Table 2, Fig. 3I). Additionally, the transversal mandibular prominence showed a moderate negative correlation with mouth breathing rates (Pearson's r = −0.473) (Table 2). Furthermore, the ratio of lower face height to mandibular width was found to have a moderate positive correlation with mouth breathing rates (Pearson's r = 0.447) (Table 2).

**Table 2:** Pearson’s correlation coefficients and 95% CIs between the 3D photographs measurements and mouth breathing rates

	The 3D photographs measurements	Pearson's correlation coefficients	95% CIs
Line	Tri-G	−0.232	(−0.557, −0.155)
	G-Sn	−0.185	(−0.202, 0.522)
	N′-Pn	0.005	(−0.226, 0.155)
	Pn-Sn	−0.287	(−0.589, 0.253)
	Li- B′	−0.129	(−0.426, −0.013)
	B′-Pog′	0.011	(−0.339, 0.287)
	Pog′-Me′	0.129	(0.003, 0.693)
	Anterior face height (N′-Me′)	−0.023	(−0.453, −0.189)
	Forehead height (Tri-N′)	0.259	(0.176, 0.753)
	Upper face height (N′-Sn)	0.182	(−0.227, 0.413)
	Lower face height (Sn-Me′)a	0.786	(0.545, 0.907)
	Mn ramus height (Tra-Go′)	0.145	(−0.273, 0.448)
	Right Mn ramus height (Tra[Rt]-Go′[Rt])	0.033	(−0.367, 0.253)
	Left Mn ramus height (Tra[Lt]-Go′[Lt])	−0.153	(−0.465, −0.090)
	Mn body length(Go′-Me′)	−0.236	(−0.654, −0.193)
	Right Mn body length (Go′[Rt]-Me′)	0.179	(0.007, 0.364)
	Left Mn body length (Go′[Lt]-Me′)	0.017	(−0.209, 0.159)
	Upper face width (Ex[Rt]-Ex[Lt])	0.298	(0.167, 0.579)
	Middle face width (Tra[Rt]-Tra[Lt])	0.036	(−0.349, 0.160)
	Lower face width (Mn width, Go′[Rt]-Go′[Lt])	0.029	(−0.349, 0.225)
	Nasal width (Al[Rt]-Al[Lt])	0.143	(0.006, 0.375)
	Philtrum width (ULP[Rt]-ULP[Lt])	0.276	(0.129, 0.686)
	Mouth width (Ch[Rt]-Ch[Lt])	−0.087	(−0.545, 0.297)
	Lower lip protrusion(Ls to SnPo')a	0.734	(0.452, 0.882)
	Upper lip protrusion (Li to SnPo')	0.255	(0.119, 0.739)
Ratio	Forehead height (Tri-N′)/Mn width(Go′[Rt]-Go′[Lt])	0.019	(−0.438, 0.337)
	Upper face height (N′-Sn)/Mn width(Go′[Rt]-Go′[Lt])	−0.015	(−0.397, 0.349)
	Lower face height (Sn-Me′)/Mn width(Go′[Rt]-Go′[Lt])a	0.447	(0.032, 0.731)
	Anterior face height (N′-Me′)/Mn width(Go′[Rt]-Go′[Lt])	0.143	(0.106, 0.782)
	Anterior face height (N′-Me′)/Zy[Rt]-Zy[Lt]	−0.279	(−0.483, 0.220)
	Interendocanthion distance (En[Rt]-En[Lt])/nasal width (Al[Rt]-Al[Lt])	−0.112	(−0.497, 0.336)
	Mouth height (ULPm-Li)/mouth width (Ch[Rt]-Ch[Lt])	−0.037	(−0.422, 0.520)
	Lower face height lower 2/3 (Li-Me′)/mn body length (average of both Go′-Me′ linear distances)	0.239	(0.019, 0.632)
	Mn ramus height (average of both Tra-Go′)/anterior face height (N′-Me′)	0.280	(0.107, 0.721)
	Upper face height (N′-Sn)/lower face height (Sn-Me′)	0.268	(0.100, 0.498)
	Total anterior face height (Tri-Me′)/Zy[Rt]-Zy[Lt]	−0.296	(−0.475, 0.334)
	Forehead height (Tri-N′)/forehead width (FT[Rt]-FT[Lt])	0.088	(−0.397, 0.442)
	Upper face height (N′-Sn)/Zy[Rt]-Zy[Lt]	0.020	(−0.297, 0.349)
	Mouth width (Ch[Rt]-Ch[Lt])/interendocanthion width (En[Rt]-En[Lt])	−0.039	(−0.248, 0.394)
	Mn width (Go′[Rt]-Go′[Lt])/interexocanthion width (Ex[Rt]-Ex[Lt])	0.228	(0.106, 0.496)
	Ex(Rt)-En(Rt)/En(Lt)-Ex(Lt)	0.257	(0.113, 0.793)
	En(Rt)-En(Lt)/En(Lt)-Ex(Lt)	0.003	(−0.495, 0.129)
	Tri-G/Sn-Me′	0.007	(−0.428, 0.223)
	G-Sn/Sn-Me′	0.025	(−0.452, 0.293)
Angle	Nasofrontal angle (G-N′-Pn)	−0.037	(−0.486, 0.253)
	Nasomental angle (N′-Pn-Pog′)	0.014	(−0.376, 0.227)
	Nasofacial angle (N′-Pn⊥G-Pog′)	0.051	(−0.426, 0.223)
	Nasolabial angle (Cm-Sn-Ls)a	−0.795	(−0.911, −0.561)
	Chin-lip angle (Li-B'-Po')a	0.773	(0.552, 0.901)
	Trans nasal prominence (Zy[Rt]-Pn-Zy[Lt])	0.268	(0.003, 0.482)
	Trans upper lip prominence (Ch[Rt]-ULPm-Ch[Lt])	0.179	(0.012, 0.498)
	Trans Mn prominence (Go′[Rt]-Pog′-Go′[Lt])a	−0.473	(−0.746, −0.065)

^a^The correlation is significant when the confidence (2-tailed) is 0.01

## Discussion

Early detection of mouth breathing is critical to reduce the prevalence of malocclusion and facilitate timely orthodontic intervention. Consequently, understanding the quantitative correlation between mouth breathing rate and malocclusion can provide valuable insights for diagnosis [33]. In this study, we developed an innovative mouth breathing monitor utilizing humidity sensors, and validated its accuracy through qualitative and quantitative assessment. Furthermore, we analyzed the correlation between mouth breath rates and three-dimensional indicators of malocclusion.

An accuracy of 101.07% indicates that the mouth breathing monitor counts the number of breathing almost precisely. In general, accuracy is defined as (TP + TN)/(TP + FP + FN + TN) (TP: True Positive, TN: True Negative, FP: False Positive, FN: False Negative) and different from accuracy we used in this study. Since TN cannot be measured in the study, we referred to the formula for calculating accuracy in the literature[24].

The monitor developed in this study showed excellent performance, achieving a sensitivity of 99.85%, which exceeds that of a tracheal sound sensor (96.06%) evaluated against PSG [19]. This high reliability can be attributed to the customized sensor holders, which were designed using a 3D-printed average face model to ensure a snug fit and enable accurate data acquisition. Furthermore, the monitor displayed a precision of 98.80% and a recall of 99.85%, reflecting a nearly balanced occurrence of false negatives (FN) and false positives (FP). This balance minimizes the impact of detection errors. Notably, the monitor maintained consistent performance across various breathing patterns and postures, with no significant differences in either accuracy or precision.

Considering that the rate and exhalation volumes of different breathing patterns varies, which may affect the response frequency and sensitivity of the modified SF films in the sensors [34]. We individually investigated and compared the accuracy, precision and recall of different breathing patterns. In the same posture, there was no significant difference in accuracy and precision between different breathing patterns. As for recall, there was no significant difference between the different breathing patterns when lying flat. However, when lying on the side, although the recall of shallow fast is significantly lower than that of shallow-slow, deep-slow, nasal, oronasal (oral) and oronasal (nasal) breathing, it reaches up to 99.17% and can be regarded as reliable. Considering that sleeping posture can affect the sensor placement and thus affecting detection [22], we recorded breathing in both flat-lying and side-lying positions to ensure the monitor's reliability. Our findings showed no significant differences in accuracy, precision, or recall across postures. This consistency may be contributed by the monitor's light weight (40 g), which minimizes positional shifts and maintains accuracy. Furthermore, compared with other devices with nasal cannulas or masks[35–39], this monitor is more user-friendly because it does not interfere sleep and therefore records real and representative breathing data during sleep[20]. Hence, the monitor developed in this study possesses significant advantages for clinical applications.

The correlation between the monitor and infrared thermography during nasal breathing was strong (−0.7/−1) [40], with a mean correlation coefficient of −0.8639. Notably, the correlation was even stronger during exhalation (−0.9332) than during inhalation (−0.8985). This disparity may be attributed to the monitor’s sensitive layer actively absorbing water molecules during exhalation, whereas their dissipation during inhalation is more passive. The linear correlation was observed between the monitor’s capacitance values and the red area ratios obtained from infrared thermography, which further validates the monitor's accuracy.

Chronic mouth breathing is associated with distinct facial characteristics known as "adenoid facies." This condition primarily arises from adenoid hypertrophy, which leads to nasal obstruction and impairs nasal breathing [12], resulting in an increased reliance on mouth breathing. Our findings support this association, revealing a strong positive correlation between adenoid volumes and mouth breathing rates (Pearson's r = 0.838). At the same time, nasal volumes, airway total volumes and nasopharynx airway volumes were negatively correlated with mouth breathing rates (Pearson's r = −0.609, −0.705, −0.784, respectively). Mouth breathing necessitates an open mouth, which can lead to a clockwise rotation of the mandible, thereby increasing the mandibular angle and lower facial height. These changes also demonstrated a strong correlation with mouth breathing rates in our study (Pearson's r = 0.742, 0.741, 0.786 respectively). Additionally, the airflow in and out through the mouth during breathing exerts pressure on the palate, resulting in a high palate and narrowed maxillary arch, which is consistent with our findings on the strong positive relationship between mouth breathing rates and maxillary canine width and palatal operculum height (Pearson's r = −0.762, 0.748). A previous study on 224 patients with skeletal Class II between ages 6 to 10, reported a slight increase in maxillary canine width following the use of a myofunctional appliance to address mouth breathing [41]. These findings suggest that early orthodontic intervention for mouth breathing can positively influence maxillofacial growth and development. In terms of soft tissue changes, our study found that nasolabial angle, lower lip protrusion and chin-lip angle were strongly correlated with mouth breathing rates (Pearson's r = −0.795, 0.734, 0.773 respectively). This aligns with previous research indicating that individuals with mouth breathing tend to exhibit a more convex nasolabial angle, lower lip protrusion and chin-lip angle [42].

**Significance and limitations of the mouth breathing monitor:** The innovative mouth breathing monitor device exhibits remarkable features, including its wearable design, lightweight construction, portability, wireless, and cost-effectiveness, making it highly suitable for both daily use and clinical application. This mouth breathing monitor is expected to become a home detection method for early identification of mouth breathing and an accurate means for rapid identification of mouth breathing at the chairside. Analysis revealed strong correlations between mouth breathing rates and various anatomical parameters, including maxillary canine width, palatal operculum height, left and right mandibular angle, lower face height, lower lip protrusion, nasolabial angle, chin-lip angle and the volumes of adenoid, nose, total airway and nasopharynx airway. Despite these advantages, the current monitor limitation lies in the presence of noise waveforms during periods of respiratory inactivity, when there was no breathing airflow passing through. Moreover, the sample size in CBCT and 3D photographs analyses is relatively small, which may limit the generalizability of the research results and the robustness of the correlation analysis. Future development will prioritize the reduction of these artifacts to enhance measurement accuracy and reliability across all operating conditions. This study is still premature and requires validation through further studies with larger samples and different clinical contexts.

## Conclusion

The wearable monitor developed in this study can simultaneously detect oral and nasal breathing qualitatively and quantitatively at high accuracy, regardless of breathing patterns and postures. Moreover, the strong correlation observed between the mouth breathing rates and malocclusion severity stresses the critical importance of early orthodontic intervention for mouth breathing. This monitor can provide evidence for early interruptive orthodontic treatment in mouth breathing, and has the potential to be a basis for the diagnosis of the mouth breathing.

## Supplementary Information

Below is the link to the electronic supplementary material.

Supplementary file1 (MP4 89014 KB)

Supplementary file2 (DOCX 5182 KB)

## Data Availability

The published article includes all data sets generated/analyzed for this study. The datasets used and analyzed during the current study are available from the corresponding author upon reasonable request.
