# Correction to: Correlation of malocclusion and mouth breathing rates from a novel monitor

**DOI:** 10.1007/s00784-026-06759-1

**Published:** 2026-02-26

**Authors:** D. N. Li, Y. Z. Tu, Z. Lu, L. Mei, F. Hua, M. H. Li, R. B. Zhu, H. He, Y. Luo, K. Qi

**Affiliations:** 1 Key Laboratory of Shaanxi Province for Craniofacial Precision Medicine Research, College of Stomatology, Xi’an Jiaotong University, 98 XiWu Road, Xi’an, 710004 China; 2 Mirco and Nano-Technology Research Center, State Key Laboratory for Manufacturing Systems Engineering (Xi’an Jiaotong University), Xi’an, 710049 China; 3 Discipline of Orthodontics, Department of Oral Sciences, Faculty of Dentistry, University of Otago, 310 Great King Street, Dunedin, 9016 New Zealand; 4 Center for Orthodontics and Pediatric Dentistry at Optics Valley Branch, School & Hospital of Stomatology, Wuhan University, Wuhan, China; 5 Department of Orthodontics, School & Hospital of Stomatology, Wuhan University, Wuhan, China

**Correction to: Clin Oral Invest**

10.1007/s00784-025-06709-3 IF: 3.1 Q1 B2

In the published paper, the authors identified missing sub-figure captions for Figs. 1, 2, and 3

The original article has been corrected.

