# Supplementary material for: Correlation of malocclusion and mouth breathing rates from a novel monitor
Source: Clin Oral Investig. 2026 Jan 7;30(1):42. doi: 10.1007/s00784-025-06709-3 (PMC12779678; doi:10.1007/s00784-025-06709-3)
Supplement: Supplementary file 2 — Supplementary file2 (DOCX 5182 KB) [file 784_2025_6709_MOESM2_ESM.docx]

*Supplementary material*

Graphical abstract: this is a graphical representation of the abstract


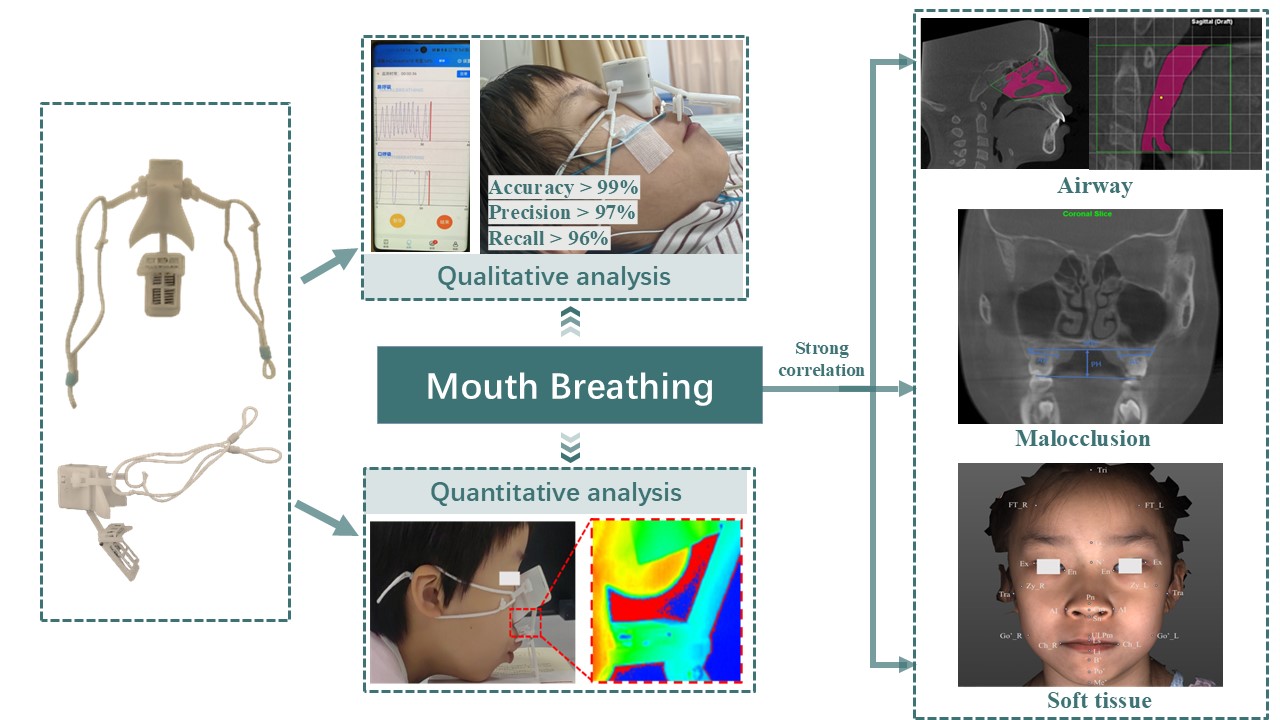


**
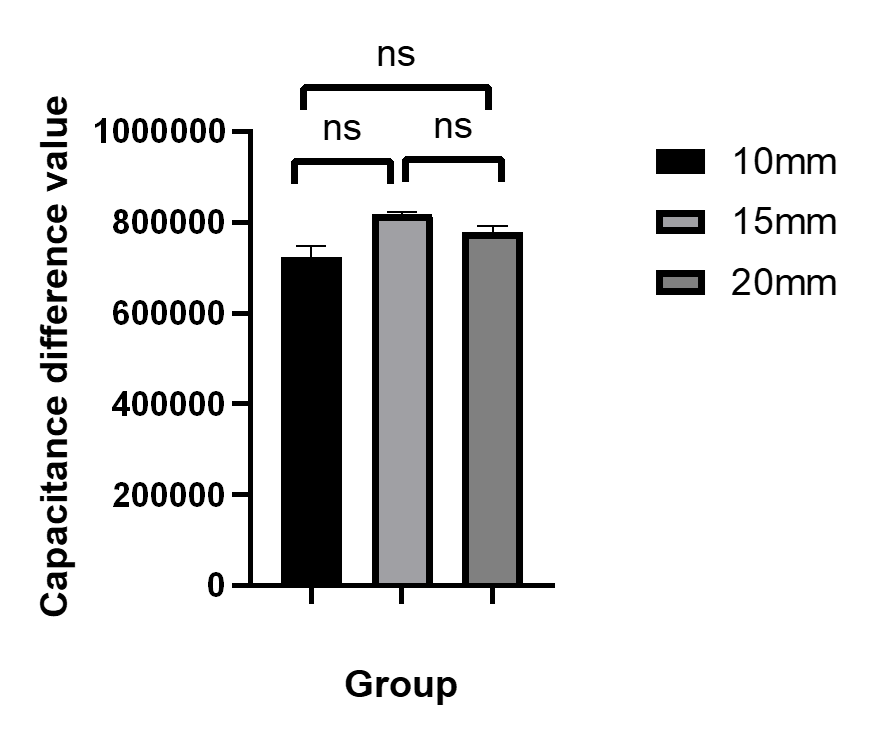
**

Supplementary Figure 1. Capacitance difference value of the mouth breathing monitor at different distances (ns, no significant difference).


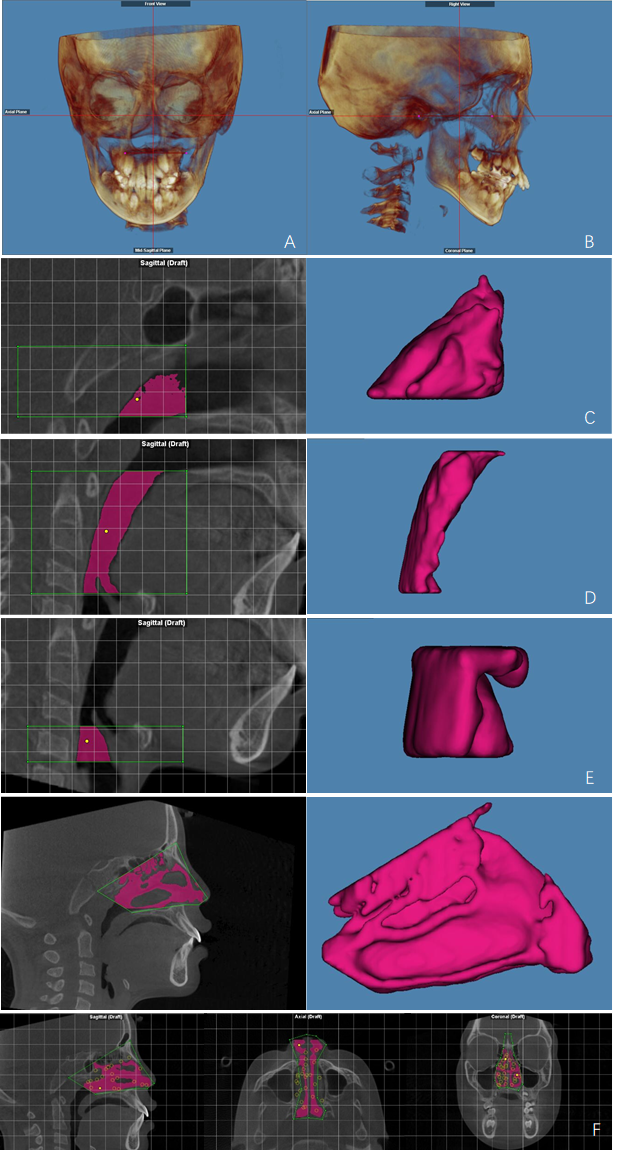
Supplementary Figure 2. The airway measurements with the Dolphin software. Skull orientation: (A) Frontal view. (B) Right sagittal view. (C) Laryngeal airway volume. (D) Oropharyngeal airway volume. (E) Nasopharyngeal airway volume. (F) The nasal landmarks.


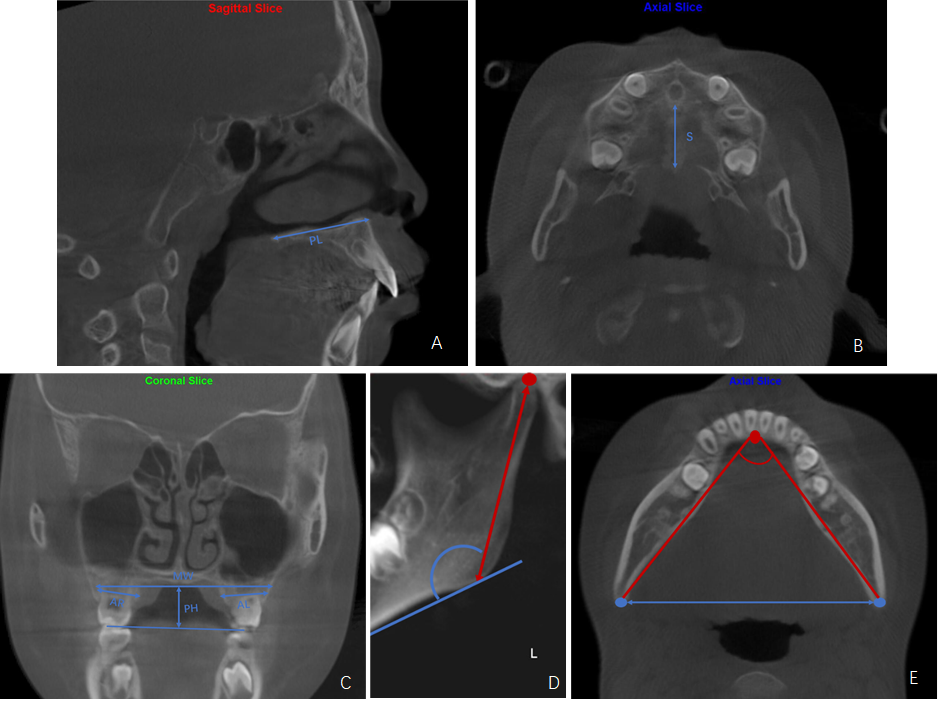


Supplementary Figure 3. The maxillary measurements with the Dolphin software. (A) Palatal length (PL) was determined in the sagittal slice. (B) The length of the mid-palatal suture (S). (C)The thickness of the alveolar crest (AL/AR), maxillary width (MW) and palatal operculum height (PH), were measured within the coronal slices. (D) Ramus height (red arrow) and mandibular angle (blue angle). Red dots represent the condylion point. (E) Mandibular width (blue arrow) and divergence of the mandible (red line). Blue dots represent mandibular gonion and red dots represent menton.


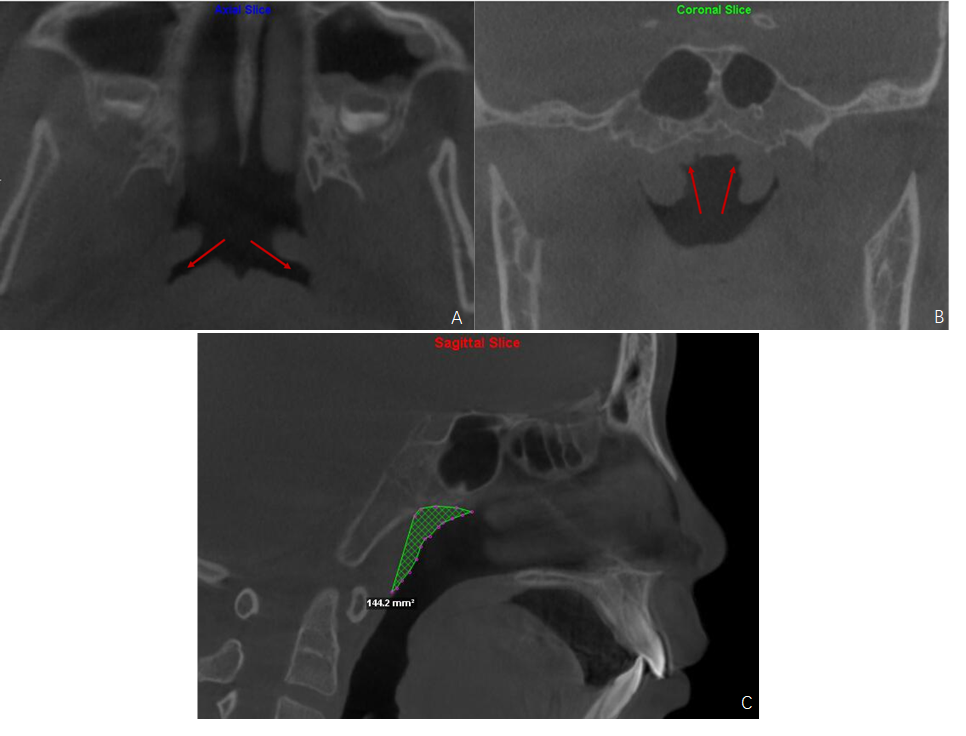


Supplementary Figure 4. CBCT slices in the Dolphin software illustrating the methodology for volumetric measurement of the adenoids. The Fossae of Rosenmüller in the axial (A) and coronal (B) slices at the arrows mark the lateral limit of the measurements. The sagittal slice (C) at midsagittal plane shows the outlined area of the surface area measurement of the adenoid. The following formula was used for calculation to estimate the volume of the adenoid gland: V = t × ∑A, where t is the section thickness and the interval of consecutive sections while ∑A is the total sectional area of the consecutive sections.


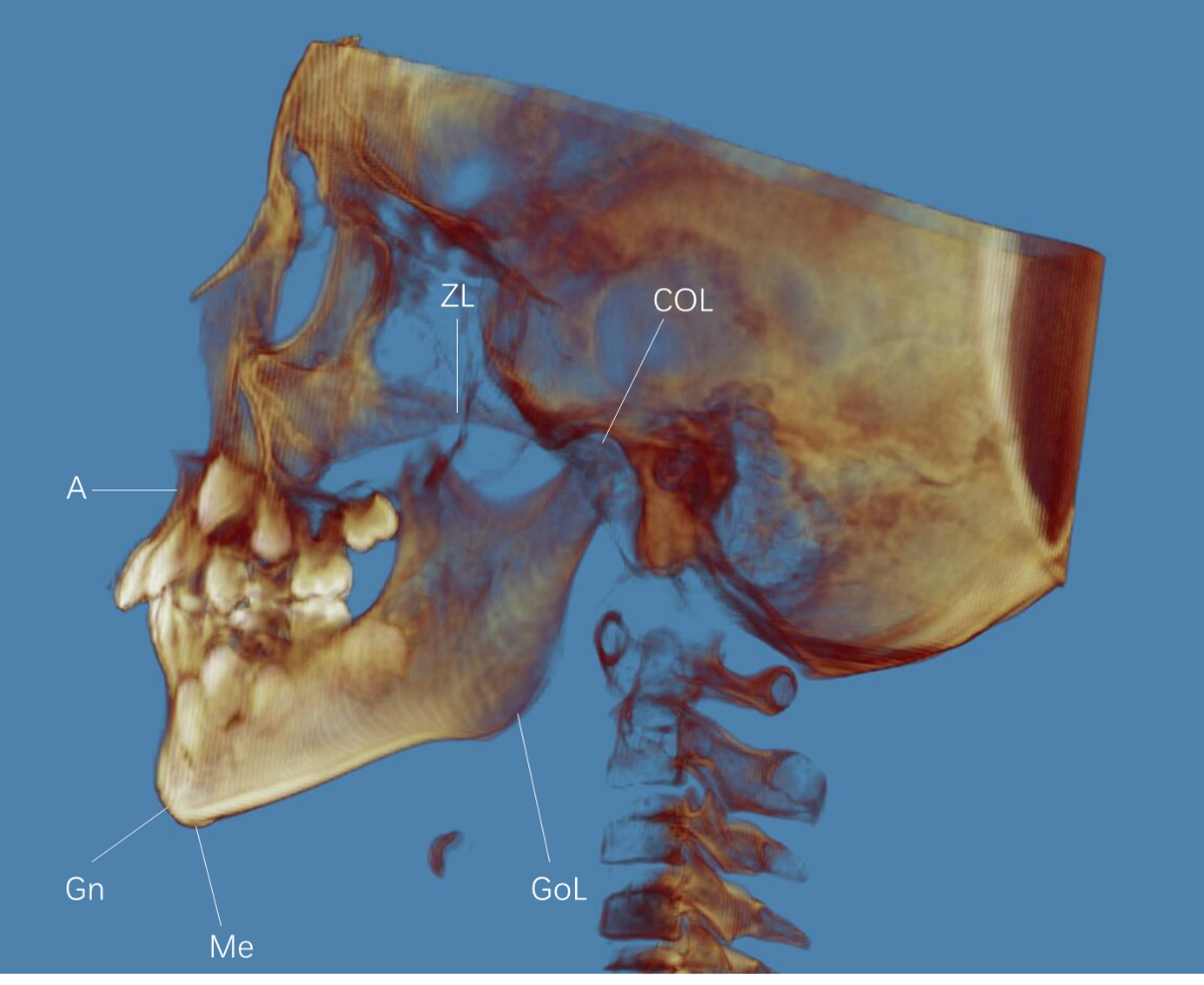


Supplementary Figure 5. Cephalometric landmarks used in the study of the 3D skull segmentations were generated from CBCT datasets of the same patient (male, 10 years old) using Dolphin 11.5 (Dolphin Imaging and Management Solutions, Chatsworth, Calif). Abbreviations and definitions of skeletal landmarks: A, point A (point of maximum midline concavity on the maxilla); Me, menton (most inferior point of mandibular symphysis); Gn, gnathion (midpoint between Pg and Me); CoL/CoR, left/right condylion (most superior point of condyle); GoL/GoR, left/right gonion (midpoint on the curvature of the angle of the mandible); ZL/ZR, left/right zygion (most lateral point of the zygomatic arch).


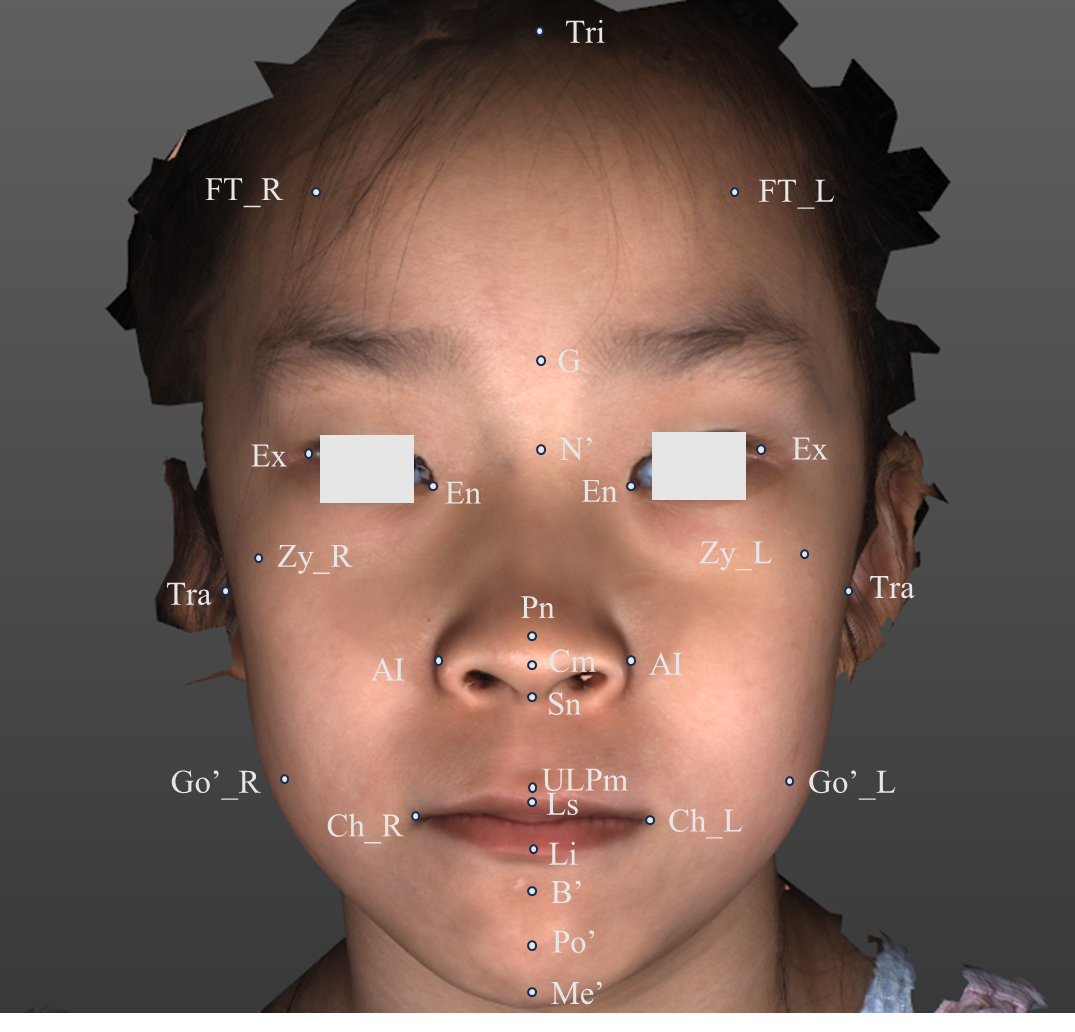


Supplementary Figure 6. The landmarks of 3D photographs: 1, N′ (soft-tissue nasion); 2, Al (nasal ala); 3, Tra (tragus); 4, Tri (trichion); 5, G (glabella); 6, En (endocanthion); 7, Ex (exocanthion); 8, Pn (pronasale); 9, Cm (nasolabial point); 10, Sn (subnasale); 11, ULPm (upper lip point); 12, Ls (labrale superior); 13, Ch (cheilion); 14, Li (labrale inferior); 15, B′ (soft-tissue B-point); 16, Pog′ (soft-tissue pogonion); 17, Me′ (soft-tissue menton); 18, FT (fronto-temporal point);  19, Go′ (soft-tissue gonion); 20, Zy (zygomatic point).

Supplementary Table 1. Histograms of accuracy, precision and recall of the number of breathing measured by mouth breathing monitor compared to PSG (*, p<0.05).

|  |  | Accuracy(%)  Mean SD | | Precision(%)  Mean SD | | Recall(%)  Mean SD | | * |
| --- | --- | --- | --- | --- | --- | --- | --- | --- |
| All |  | 101.07 | 5.21 | 98.80 | 3.70 | 99.85 | 2.08 |  |
| Posture  (all breathing patterns) | Lying | 101.04 | 4.82 | 98.67 | 4.06 | 99.79 | 2.54 |  |
|  | Side-lyijng | 101.10 | 5.59 | 98.93 | 3.30 | 99.90 | 1.49 |  |
| Breathing patterns  (lying) | Slow-fast | 100.71 | 2.47 | 99.05 | 1.97 | 99.71 | 1.07 |  |
|  | Shallow | 102.27 | 8.32 | 97.60 | 5.87 | 100.71 | 2.86 |  |
|  | Deep-fast | 101.22 | 4.35 | 98.50 | 3.54 | 99.56 | 1.36 |  |
|  | Deep-slow | 100.93 | 2.91 | 99.15 | 2.63 | 100.00 | 0.00 |  |
|  | Nasal | 100.61 | 2.88 | 98.06 | 6.59 | 99.80 | 1.08 |  |
|  | Oronasal(nasal) | 102.09 | 4.29 | 98.54 | 4.26 | 100.12 | 2.06 |  |
|  | Oronasal(oral) | 100.50 | 7.13 | 98.75 | 3.34 | 98.82 | 5.58 |  |
|  | oral | 99.98 | 2.66 | 99.69 | 1.64 | 99.64 | 1.89 |  |
| Breathing patterns  (side-lying) | Slow-fast | 99.86 | 2.86 | 99.34 | 1.72 | 99.17 | 1.91 |  |
|  | Shallow | 104.08 | 12.65 | 97.60 | 5.87 | 100.71 | 2.86 |  |
|  | Deep-fast | 100.59 | 2.63 | 99.35 | 2.26 | 99.88 | 0.65 |  |
|  | Deep-slow | 100.81 | 3.08 | 99.28 | 2.73 | 100.00 | 0.00 |  |
|  | Nasal | 100.10 | 1.76 | 99.75 | 1.35 | 100.00 | 0.00 |  |
|  | Oronasal(nasal) | 102.61 | 5.47 | 98.04 | 4.38 | 100.00 | 0.00 |  |
|  | Oronasal(oral) | 101.10 | 3.27 | 98.73 | 3.20 | 100.00 | 0.00 |  |
|  | oral | 99.72 | 3.58 | 99.31 | 2.14 | 99.42 | 2.14 |  |

Supplementary Table 2. Inter- and intraclass correlation coefficients for CBCT measurements.

|  | The CBCT measurements | Interclass coefficients | Intraclass coefficients |
| --- | --- | --- | --- |
| Line | Nasopharyngeal heights | 0.908 | 0.884 |
|  | Oropharyngeal heights | 0.910 | 0.968 |
|  | Laryngeal heights | 0.953 | 0.867 |
|  | Toltal heights | 0.895 | 0.924 |
|  | the length of the mid-palatal suture | 0.867 | 0.935 |
|  | Palatal length (PL) | 0.879 | 0.867 |
|  | the thickness of the left alveolar crest | 0.877 | 0.945 |
|  | the thickness of the right alveolar crest | 0.949 | 0.875 |
|  | maxillary canines width | 0.856 | 0.885 |
|  | palatal operculum height | 0.896 | 0.972 |
|  | Mandibular width | 0.942 | 0.974 |
|  | CL.GoL.Me | 0.867 | 0.965 |
|  | CR.GoR.Me | 0.887 | 0.872 |
|  | CL-CR | 0.942 | 0.978 |
|  | GoL-GoR | 0.857 | 0.889 |
|  | ZL-ZR | 0.893 | 0.915 |
|  | CL-GoL | 0.968 | 0.957 |
|  | CR-GoR | 0.872 | 0.849 |
|  | GoL-Me | 0.894 | 0.925 |
|  | GoR-Me | 0.879 | 0.895 |
|  | CL-A | 0.891 | 0.941 |
|  | CR-A | 0.873 | 0.948 |
|  | CL-Gn | 0.896 | 0.945 |
|  | CR-Gn | 0.907 | 0.938 |
| Angle | Left mandibular angle | 0.868 | 0.884 |
|  | Right mandibular angle | 0.871 | 0.883 |
|  | Divergence of the mandible | 0.945 | 0.952 |
| Area | Minimum areas of nasopharynx | 0.892 | 0.948 |
|  | Minimum oropharyngeal areas | 0.931 | 0.879 |
|  | Minimum laryngeal areas | 0.898 | 0.942 |
| Volume | Nasal volumes | 0.869 | 0.837 |
|  | Nasopharyngeal volumes | 0.874 | 0.958 |
|  | Oropharyngeal volumes | 0.909 | 0.951 |
|  | Laryngeal volumes | 0.894 | 0.948 |
|  | Adenoid voulmes | 0.883 | 0.878 |
|  | Total airway volume | 0.875 | 0.853 |

Supplementary Table 3. Inter- and intraclass correlation coefficients for 3D photograph measurements.

|  | The 3D photographs measurements | Interclass coefficients | Intraclass coefficients |
| --- | --- | --- | --- |
| Line | Tri-G | 0.932 | 0.957 |
|  | G-Sn | 0.885 | 0.922 |
|  | N′-Pn | 0.895 | 0.926 |
|  | Pn-Sn | 0.928 | 0.859 |
|  | Li-B | 0.929 | 0.926 |
|  | B′-Pog′ | 0.911 | 0.897 |
|  | Pog′-Me′ | 0.929 | 0.893 |
|  | Anterior face height (N′-Me′) | 0.923 | 0.889 |
|  | Forehead height (Tri-N′) | 0.959 | 0.893 |
|  | Upper face height (N′-Sn) | 0.882 | 0.927 |
|  | Lower face height (Sn-Me′) | 0.886 | 0.907 |
|  | Mn ramus height (Tra-Go′) | 0.945 | 0.884 |
|  | Right Mn ramus height (Tra[Rt]-Go′[Rt]) | 0.933 | 0.967 |
|  | Left Mn ramus height (Tra[Lt]-Go′[Lt]) | 0.953 | 0.946 |
|  | Mn body length(Go′-Me′) | 0.936 | 0.954 |
|  | Right Mn body length (Go′[Rt]-Me′) | 0.879 | 0.934 |
|  | Left Mn body length (Go′[Lt]-Me′) | 0.917 | 0.928 |
|  | Upper face width (Ex[Rt]-Ex[Lt]) | 0.898 | 0.916 |
|  | Middle face width (Tra[Rt]-Tra[Lt]) | 0.863 | 0.910 |
|  | Lower face width (Mn width, Go′[Rt]-Go′[Lt]) | 0.929 | 0.891 |
|  | Nasal width (Al[Rt]-Al[Lt]) | 0.943 | 0.898 |
|  | Philtrum width (ULP[Rt]-ULP[Lt]) | 0.876 | 0.868 |
|  | Mouth width (Ch[Rt]-Ch[Lt]) | 0.887 | 0.927 |
|  | Lower lip protrusion( Ls to SnPo') | 0.934 | 0.964 |
|  | Upper lip protrusion (Li to SnPo') | 0.955 | 0.879 |
| Ratio | Forehead height (Tri-N′)/Mn width(Go′[Rt]-Go′[Lt]) | 0.901 | 0.896 |
|  | Upper face height (N′-Sn)/Mn width(Go′[Rt]-Go′[Lt]) | 0.915 | 0.949 |
|  | Lower face height (Sn-Me′)/Mn width(Go′[Rt]-Go′[Lt]) | 0.877 | 0.913 |
|  | Anterior face height (N′-Me′)/Mn width(Go′[Rt]-Go′[Lt]) | 0.943 | 0.872 |
|  | Anterior face height (N′-Me′)/Zy[Rt]-Zy[Lt] | 0.879 | 0.943 |
|  | Interendocanthion distance (En[Rt]-En[Lt])/nasal width (Al[Rt]-Al[Lt]) | 0.940 | 0.936 |
|  | Mouth height (ULPm-Li)/mouth width (Ch[Rt]-Ch[Lt]) | 0.937 | 0.925 |
|  | Lower face height lower 2/3 (Li-Me′)/mn body length (average of both Go′-Me′ linear distances) | 0.939 | 0.919 |
|  | Mn ramus height (average of both Tra-Go′)/anterior face height (N′-Me′) | 0.962 | 0.968 |
|  | Upper face height (N′-Sn)/lower face height (Sn-Me′) | 0.898 | 0.943 |
|  | Total anterior face height (Tri-Me′)/Zy[Rt]-Zy[Lt] | 0.926 | 0.934 |
|  | Forehead height (Tri-N′)/forehead width (FT[Rt]-FT[Lt]) | 0.960 | 0.949 |
|  | Upper face height (N′-Sn)/Zy[Rt]-Zy[Lt] | 0.920 | 0.934 |
|  | Mouth width (Ch[Rt]-Ch[Lt])/interendocanthion width (En[Rt]-En[Lt]) | 0.939 | 0.943 |
|  | Mn width (Go′[Rt]-Go′[Lt])/interexocanthion width (Ex[Rt]-Ex[Lt]) | 0.882 | 0.946 |
|  | Ex(Rt)-En(Rt)/En(Lt)-Ex(Lt) | 0.937 | 0.913 |
|  | En(Rt)-En(Lt)/En(Lt)-Ex(Lt) | 0.903 | 0.894 |
|  | Tri-G/Sn-Me′ | 0.870 | 0.923 |
|  | G-Sn/Sn-Me′ | 0.925 | 0.934 |
| Angle | Nasofrontal angle (G-N′-Pn) | 0.937 | 0.948 |
|  | Nasomental angle (N′-Pn-Pog′) | 0.914 | 0.950 |
|  | Nasofacial angle (N′-Pn⊥G-Pog′) | 0.851 | 0.938 |
|  | Nasolabial angle (Cm-Sn-Ls) | 0.895 | 0.911 |
|  | Chin-lip angle ( Li-B'-Po') | 0.946 | 0.952 |
|  | Trans nasal prominence (Zy[Rt]-Pn-Zy[Lt]) | 0.858 | 0.942 |
|  | Trans upper lip prominence (Ch[Rt]-ULPm-Ch[Lt]) | 0.951 | 0.948 |
|  | Trans Mn prominence (Go′[Rt]-Pog′-Go′[Lt]) | 0.947 | 0.965 |
